# Effects of Two Different Training Programs on Cardiometabolic Health, Body Composition and Irisin in Middle Age Obese Males: A Pilot Study

**DOI:** 10.3390/life16040657

**Published:** 2026-04-13

**Authors:** Mattia D’Alleva, Marta Mallardo, Nicola Giovanelli, Francesco Graniero, Federica Fiori, Michela Marinoni, Maria Parpinel, Lara Mari, Enrico Rejc, Simone Zaccaron, Jacopo Stafuzza, Stefano Lazzer, Aurora Daniele, Ersilia Nigro

**Affiliations:** 1 Department of Medicine, University of Udine, 33100 Udine, Italy; mattia.dalleva@uniud.it (M.D.); info@nexthillcoaching.com (N.G.); federica.fiori@uniud.it (F.F.); michela.marinoni@uniud.it (M.M.); maria.parpinel@uniud.it (M.P.); lara.mari@uniud.it (L.M.); enrico.rejc@uniud.it (E.R.); simone.zaccaron@uniud.it (S.Z.); jacopo.stafuzza@uniud.it (J.S.); stefano.lazzer@uniud.it (S.L.); 2 School of Sport Sciences, University of Udine, 33100 Udine, Italy; 3 Department of Theoretical and Applied Sciences, eCampus University, Novedrate, 22060 Como, Italy; 4 Dipartimento di Medicina Molecolare e Biotecnologie Mediche, Università degli Studi “Federico II”, Via Pansini 5, 80131 Napoli, Italy; marta.mallardo@unipegaso.it (M.M.); ersilia.nigro@unicampania.it (E.N.); 5 CEINGE-Biotechnologies Advances Franco Salvatore S.c.a r.l., Via G. Salvatore 486, 80145 Naples, Italy; 6 Physical Exercise Prescription Center, Azienda Sanitaria Universitaria Friuli Centrale, Gemona del Friuli, 33013 Udine, Italy; graniero.francesco@libero.it; 7 Department of Neurosciences, Biomedicine and Movement Sciences, University of Verona, 37129 Verona, Italy; 8 Dipartimento di Scienze e Tecnologie Ambientali, Biologiche, Farmaceutiche, Università della Campania “Luigi Vanvitelli”, Via Vivaldi 43, 81100 Caserta, Italy

**Keywords:** exercise, obesity, body composition, irisin, cardiometabolic health

## Abstract

Obesity is a chronic disease characterized by excessive fat accumulation. Irisin, released during exercise, regulates energy metabolism and may contribute to exercise-induced metabolic adaptations. This study aimed to compare the effects of 24 weeks of two different training programs on body composition, physical capacities, and irisin levels in male adults with obesity, and to investigate the relationship between irisin and metabolic parameters. Thirteen male adults with obesity were randomly assigned to polarized (POL) or threshold (THR) training programs. Anthropometric measurements, physical capacity parameters, serum and salivary samples were collected before (T0) and after the training period (T1). Irisin levels were measured by ELISA. After training, body composition significantly improved, with reductions in body mass and body mass index, and an increase in fat-free mass. Maximal oxygen consumption (V’O_2_max) significantly increased, while a decrease in HRmax indicated improved cardiac efficiency. Although serum and salivary irisin levels did not significantly increase overall, a trend toward increased irisin was observed in the THR group. Furthermore, serum irisin at T1 positively correlated with V’O_2_ at the respiratory compensation point (*p* = 0.019), and V’O_2_max (*p* = 0.031). Both POL and THR training programs significantly improved body composition and cardiometabolic fitness after 24 weeks. The positive association of irisin with aerobic fitness parameters suggests that irisin may reflect physiological adaptations to exercise.

## 1. Introduction

Obesity currently represents one of the most serious health challenges worldwide [1]. In Italy, the global obesity observatory report obesity in around 12% of the total population [2], with a higher incidence in men. Physical activity represents one of the most effective interventions to obtain multiple beneficial effects on health, including the reduction in obesity [3]; however, the World Health Organization reported that less than 50% of the population reaches sufficient physical activity levels [4]. In this context, aerobic physical exercise is an important component of a healthy lifestyle, so much so that it represents the most valid non-pharmacological approach for the treatment of obesity and related disorders [5]. Moderate-intensity continuous training (MICT) and high-intensity interval training (HIIT) are the most common types of aerobic physical exercise used [6]. MICT involves 20–60 min sessions at <80% heart rate max (HRmax) or 49–75% at maximal oxygen uptake (V’O_2_max), while HIIT consists of 1–4 min intervals at ≥85% HRmax or ≥80% V’O_2_max [7]. HIIT has been shown to improve cardiorespiratory fitness (CRF) and body composition equal or greater than MICT in people with obesity over short periods (4–16 weeks) [8]. However, recent studies suggest that a polarized (POL) training approach, combining high- and moderate-intensity exercise, is equally or more effective in enhancing physiological parameters and improving body composition than either HIIT or MICT alone [9,10].

The effectiveness of different exercise modalities in people with obesity extends beyond cardiometabolic improvements to the molecular level, influencing the endocrine function of skeletal muscle. Specifically, aerobic exercise stimulates muscle cells to secrete irisin, a hormone of 112 amino acids (aa), which is generated by cleavage of the transmembrane protein FNDC-5 (also known as fibronectin type III repeat-containing protein 2, FRCP-2 [11]. In humans, irisin has garnered attention for its role in increasing energy expenditure by stimulating the browning of white adipose tissue, promoting thermogenesis, enhancing mitochondrial biogenesis and improving oxidative metabolism, thereby contributing to enhanced insulin sensitivity and glucose tolerance [12]. However, the relationship between exercise and irisin remains one of the most controversial topics in this field. Previous studies have investigated the association between serum irisin levels and obesity, but the results are inconsistent [13,14,15,16]. The type and intensity of exercise affect irisin up-regulation. D’Amuri et al. demonstrated that either HIIT or MICT reduced irisin levels in response to 12 weeks of exercise [17]. On the contrary, another work reported that HIIT showed a significant increase in irisin, while moderate aerobic exercise did not induce any change [18]. Therefore, considering the limited evidence currently available, the specific impact of exercise intensity and volume on irisin production in individuals with obesity remains unclear [19,20]. At the same time, to our knowledge, no studies have investigated the combined effects of HIIT and MICT on irisin response, despite evidence suggesting that this training approach may induce improvements in body composition and physical capacity that are similar or even superior to HIIT or MICT performed alone [9,10].

Therefore, the present study aimed to investigate the effects of 24 weeks of two different training programs, polarized (POL) and threshold (THR), on body composition, physical capacities, and irisin levels in obese adult males.

Based on previous evidence suggesting that exercise modulates irisin release, but with inconsistent findings depending on training intensity and modality, we formulated the following hypotheses: first, we hypothesized that long-term aerobic training would induce an increase in circulating and salivary irisin levels; second, we hypothesized that the two training modalities would differently impact the magnitude and direction of irisin modulation, with a potentially greater response in the THR group due to the higher proportion of exercise performed at intensities above the gas exchange threshold.

Additionally, we hypothesized that irisin levels would be associated with training-induced adaptations, particularly improvements in aerobic fitness parameters, such as V’O_2_max, supporting a potential role of irisin as a biomarker of exercise-induced physiological adaptations. We tested irisin levels in serum as well as in saliva; the latter biological sample represents a potential advantage, as saliva is a non-invasive biological fluid that allows easier, stress-free, and repeated sampling compared to blood collection.

## 2. Materials and Methods

### 2.1. Participants, Training Programs and Sample’s Collection

**Figure 1:**
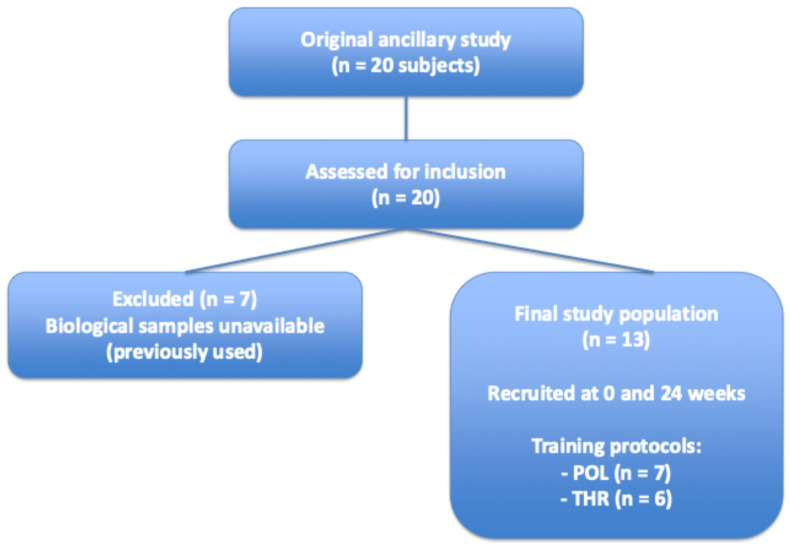
Flowchart of the study population selection.

As shown in the study flowchart (Figure 1), thirteen Caucasian male subjects with obesity, part of a previous ancillary study including twenty subjects, were recruited for the present study from the School of Sport Sciences of the University of Udine (mean age 40.8 ± 6.2 years; mean BMI: 32.0 ± 3.0 kg m^−^^2^) [9]. Of the original twenty participants, only thirteen attended the laboratory session for blood sample analyses. The inclusion criteria required body mass to be stable over the two months preceding the study. All participants were free from any diagnosed cardiovascular, respiratory, neurological, musculoskeletal, metabolic, or endocrine conditions, and none reported regular use of medications or substances known to interfere with energy metabolism [9]. The study was approved by the Ethics Committee of the Friuli-Venezia-Giulia Region (Italy) (P.N. 1764) and conducted according to the ethical principles of the Declaration of Helsinki. Informed consent was obtained from all participants before the study began. After an eight-week pre-intervention period, participants were randomly assigned to either a polarized (POL, n = 10) or threshold (THR, n = 10) training group and followed a 24-week weight management program. Both groups trained under real-life conditions and completed identical pre- (PRE) and post- (POST) assessments, including anthropometry, body composition, and a graded exercise test to determine ventilatory thresholds and V’O_2_max. The training program consisted of three weekly sessions of walking and/or running and was structured into three eight-week macrocycles (3 + 1 mesocycles) with linear periodization. Training load (TL) increased similarly in both groups (~30% from macrocycle 1 to 2 and ~10% from 2 to 3), with a 30% reduction during recovery weeks. Importantly, TL was matched between POL and THR using TRIMP. The key difference between groups was the training intensity distribution (TID), calculated every eight weeks using a three-zone model based on speed at gas exchange threshold (GET), respiratory compensation point (RCP), and V’O_2_max: Z1 (<GET), Z2 (GET–RCP), and Z3 (>RCP). The POL group followed a polarized model (Z1 > Z3 > Z2), while the THR group emphasized threshold training (greater time in Z2). A typical training week for the POL group consisted of two low-intensity sessions of 60 min each performed in Z1, along with one weekly interval training session (e.g., 8 × 1 min in Z3). In contrast, a typical week for the THR group included one 60 min session in Z1 and two additional weekly sessions characterized by a combination of work in Z2 and Z3, for example, 3 × (2 min in Z1 + 6 min in Z2 + 1 min in Z3). Training data (duration, time in zones, rate of perceived exertion, RPE) were recorded via wearable devices and verified by trainers. Intensity was adjusted when heart rate decreased by ≥5 bpm across sessions. At the end of the intervention, participants completed a running challenge (half marathon, 30 km, or marathon) under medical supervision.

During the 24 weeks of training, all participants recorded their workouts using an online training diary Polar Flow (Polar Electro Oy, Finland) or Gamin Connect (Garmin, Olathe, USA). The training was checked online registering the training sessions: duration, time spent in each endurance training zone, and RPE using the Borg 6–20 Scale. For maintaining the intensity prescribed, the speed in each zone was increased when the mean HR decreased by 5 bpm for two consecutive training sessions. Before (T0) and after the end (T1) the training period, blood and saliva samples were collected after a 12 h overnight fasting period, as previously described [21].

### 2.2. Anthropometric, Irisin and Adiponectin Measurements

The anthropometric and biochemical data of the study participants are presented in Table 1. Body mass (BM) was assessed using a manual weighing scale (with an accuracy of 0.1 kg) (Seca 709, Hamburg, Germany). Stature was measured with a wall-mounted height board. The BMI was calculated as BM (kg) divided by stature (m^2^). Waist circumference (WC) and hip circumference (HC) were measured following the method outlined by Kagawa et al. [22]. Body composition was evaluated through bioelectrical impedance analysis (BIA) using the Human IM Plus device (DS 171 Dieto-system, Milan, Italy). Fat mass (FM) and fat-free mass (FFM) values were determined using the equations provided by Gray et al. [23].

Irisin concentrations were measured in both serum and saliva using a commercial ELISA kit (Human Irisin ELISA Kit, ab285295, Abcam, Cambridge, UK), according to the manufacturer’s instructions. The assay range was 0.001 µg/mL –5 mg/mL, with a sensitivity of 1 ng/mL. Interassay CV < 10%, intraassay CV < 10%. Although this assay is primarily validated for serum samples, its application to saliva has been previously reported [8,24]. Salivary irisin has been proposed as a non-invasive alternative to blood sampling and has been shown to reflect circulating levels under specific conditions [24].

We acknowledge that salivary measurements may be influenced by factors such as flow rate, hydration status, and circadian variability. To minimize these effects, all saliva samples were collected under standardized conditions, after an overnight fast and at the same time of day, two to seven days after the final training session, as previously described [25].

The concentration of total adiponectin was measured by an enzyme-linked immunosorbent assay (ELISA) method using an in-house produced polyclonal antibody designed against the human Acrp30 amino acid region (H2N-ETTTQGPGVLLPLPKG-COOH), as previously reported [21].

### 2.3. Graded Exercise Test (GRAD)

A graded exercise test was performed on a 400 m track to assess the physical capacities of each participant, including V’O_2_max, HRmax, and ventilatory thresholds. Participants were instructed to maintain their habitual diet and to refrain from consuming alcohol and caffeine for 24 h before testing and from large meals for at least 3 h before testing. During the test, participants followed a researcher on a bike who controlled the pace. Each stage lasted 1 min, with the speed increasing by 0.5 km h^−1^ every minute until volitional exhaustion. Oxygen uptake (V’O_2_), carbon dioxide production (V’CO_2_), and heart rate (HR) were measured using a wearable metabolic unit (K5; Cosmed, Italy) and a chest strap (Garmin HRMrun, Olathe, USA). Prior to each test, the volume and gas analyzers were calibrated with a 3 L calibration syringe and calibration gas (16.00% O_2_ and 5.00% C’O_2_), respectively. The ventilatory threshold, GET and RCP were determined using the V-slope method. V’O_2_max was calculated as the average 30 s V’O_2_ based on the following criteria: (i) a plateau in V’O_2_ (increase <150 mL min^−1^), (ii) a Respiratory Exchange Ratio (RER) > 1.1, and (iii) ≥90% of the theoretical HRmax.

### 2.4. Dietary Habits

Participants were invited to collect a four-day dietary record (4-dDR), collecting food and beverage consumption of two weekdays and two weekend days, with instructions on how to record the type and portion size of the foods consumed to calculate daily energy and macronutrient intake, as previously described [25].

### 2.5. Statistical Analysis

Data analysis was performed using GraphPad Prism (version 10.0.0), with statistical significance set at *p* < 0.05. Results are presented as means ± standard deviations (SDs), or medians and interquartile ranges in the case of non-normally distributed data. The normality of data distribution was assessed using the Shapiro–Wilk test. Mauchly’s test was employed to verify sphericity, and in cases where sphericity assumptions were violated, the Greenhouse–Geisser correction was applied. Data were presented for the whole sample following the results of the two-way ANOVA (group × time), which showed no significant group effects or group × time interactions for the primary outcomes [9]. This approach was adopted to ensure consistency with the statistical model and to provide an overall description of changes over time within the study population, which is part of a larger research project [9]. Thus, paired *t*-tests were used to assess within-group changes in anthropometric characteristics, body composition, V’O_2_max, ventilatory thresholds, and training variables. A two-way ANOVA, including the between-subjects factor of the training model (POL and THR groups) and the within-subjects factor of time (T0 vs. T1, i.e., repeated measured analysis), was employed to evaluate differences in Irisin concentrations between groups. For the analysis of salivary irisin, we used data from five participants in the THR group, compared to six participants in the serum irisin analysis. For ANOVA analyses, effect sizes were expressed as partial eta squared (ηp^2^) Cohen et al., 1988). Thresholds for interpretation were considered small ≥ 0.01, medium ≥ 0.06, and large ≥ 0.14. Finally, the Cohen ES was calculated for detecting the magnitude of within-group changes [26]. An ES less than 0.20 was considered trivial, values between 0.20 and 0.49 were classified as small, between 0.50 and 0.79 as moderate, and >0.80 was considered large. Bivariate correlations were determined using Pearson’s correlation coefficient for normally distributed data and Spearman’s rank correlation coefficient for non-normally distributed data.

## 3. Results

### 3.1. Anthropometric Characteristics, Cardiometabolic/Fitness Parameters and Irisin of Obese Subjects Following Exercise Training

The present study is an ancillary study to a previous one [21]. The anthropometric and biochemical parameters of the study participants are shown in Table 1. At the end of the program (T1), compared to baseline (T0), body composition significantly improved through reductions in body mass (99.0 ± 10.8 vs. 94.6 ± 11.5 kg), body mass index (32.0 ± 3.0 vs. 30.6 ± 3.0 kg), and increases in fat-free mass (65.2 ± 3.9 vs. 68.6 ± 4.2 kg) (see Table 1). Serum adiponectin values (μg/mL) were 22.3 ± 4.5 at T0 vs. 27.0 ± 2.6 at T1 (*p* < 0.001), while salivary adiponectin changed from 17.6 ± 7.5 to 22.8 ± 10.0 ng/mL at T0 and T1, respectively (*p* = 0.005).

Cardiometabolic fitness parameters are shown in Table 2. V’O_2_max statistically increased and HR max statistically decreased at T1 compared to T0, suggesting an increased in cardiometabolic and fitness parameters at the end of the training program. A complete overview of the physiological parameters measured before and after the different training sessions can be found in Table 2.

Daily energy and macronutrient intake before (T0) and after 24 weeks (T1) of weight management program did not change (Table 3).

### 3.2. Irisin Modulation and Correlation with Fitness Parameters at the End of the Training Program

**Figure 2:**
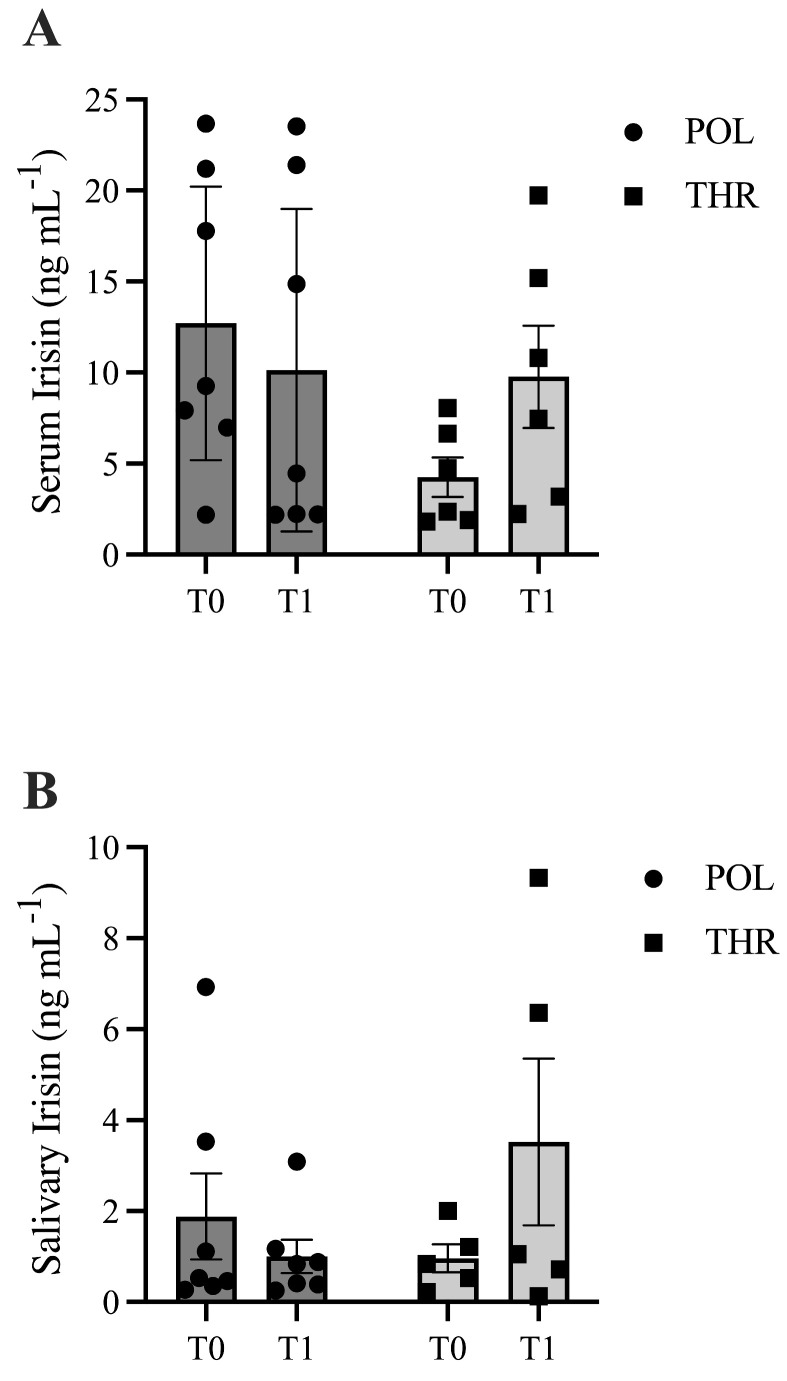
Graphical representation of serum (**panel A**) and salivary irisin (**panel B**) levels before (T0) and after 24 weeks (T1) of the weight management program, divided according to the different training programs (POL and THR) (**A**,**B**).

Considering all participants, serum and salivary irisin levels did not increase at T1 (Table 1). When participants were stratified according to the training program (POL vs. THR), a trend toward a group × time interaction was observed for serum irisin (*p* = 0.093, F (1,11) = 3.194, ηp^2^ = 0.225). Specifically, an increase was detected only in the THR group (4.3 ± 2.7 vs. 9.8 ± 6.9 ng/mL, ES = 1.05, large), although post hoc analysis did not reach statistical significance (*p* = 0.106). A similar pattern was observed for salivary irisin, where a near-significant group × time interaction emerged (*p* = 0.051, F(1,10) = 3.868, ηp^2^ = 0.279), with increases observed only in the THR group (0.96 ± 0.69 vs. 2.19 ± 2.52 ng/mL, ES = 0.67, moderate-to-large), despite non-significant post hoc comparisons (*p* = 0.161) (Figure 2, panels A and B).

In addition, considering the delta values of both serum and salivary irisin, we observed a tendency toward a decrease in the POL group for serum irisin, whereas in the THR group there was an increase (−2.9 ± 8.5 vs. 4.3 ± 7.0 ng/mL, respectively; *p* = 0.089). Similarly, salivary irisin tended to decrease in the POL group, while it tended to increase in the THR group (−1.0 ± 2.1 vs. 2.6 ± 3.9 ng/mL, respectively; *p* = 0.093).

### 3.3. Correlations

Correlation analyses were considered exploratory and were not adjusted for multiple comparisons. Correlation analysis between irisin and the anthropometric parameters in subjects at T0 are shown in Supplementary Table S1; we failed to find any correlation at T0 (Supplementary Table S2).

**Figure 3:**
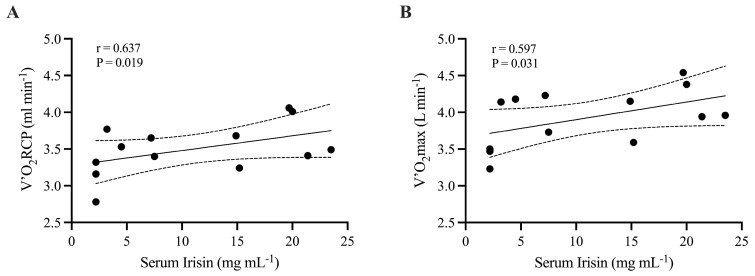
Correlation analysis between serum irisin at T1 and V’O_2_RCP (**panel A**) and V’O_2_max (**panel B**).

Similarly, we failed to find any correlation at T0 between irisin and the physical capacities of participants (Supplementary Table S2), while at T1, irisin directly correlates with V’O_2_RCP (r = 0.637, *p* = 0.019) (Figure 3, panel A) and V’O_2_max (r = 0.597, *p* = 0.031) (Figure 2, panel B). No significant correlations were found for salivary irisin.

## 4. Discussion

The main findings of this study are as follows: (a) 24 weeks of structured aerobic training based on POL and THR intensity distribution models significantly improved body composition and cardiorespiratory fitness in adult males with obesity; (b) while irisin concentrations did not change significantly in the overall sample, a trend toward a group × time interaction was observed, with both circulating and salivary irisin levels increasing only in the THR group; and (c) irisin levels at the end of the 24-week training period were significantly associated with key indicators of aerobic fitness, V’O_2_RCP and V’O_2_max.

The first result showed that both training programs resulted in a significant improvement in body composition, characterized by reductions in BM and BMI and preservation or modest increases in FFM. These findings are consistent with previous observations from our group and others, demonstrating that structured aerobic training programs combining moderate- and higher-intensity exercise can effectively improve body composition and metabolic health in obese individuals [21,24]. Similarly, improvements in V’O_2_max and ventilatory thresholds confirm the effectiveness of long-term aerobic training in enhancing cardiorespiratory fitness in this population. V’O_2_max is widely recognized as a key indicator of aerobic power and overall cardiovascular health [27], and its increase reflects both central adaptations (e.g., improved cardiac output) and peripheral adaptations (e.g., greater mitochondrial function and capillary density) [28]. Recent evidence has confirmed that structured aerobic exercise consistently leads to meaningful enhancements in V’O_2_max across a broad range of study populations and training durations, highlighting its role in improving oxygen utilization and metabolic function [29,30].

The second important finding was that, although no statistically significant increase in circulating or salivary irisin levels was observed when considering the entire cohort, a consistent trend toward an increase in irisin concentrations was detected, particularly in the THR group. Although no statistically significant changes in circulating or salivary irisin levels were observed, descriptive data suggested a possible increase in the THR group. This observation should be interpreted cautiously, as the limited sample size likely reduced the statistical power to detect subtle effects. In addition, the known inter-individual variability in irisin response might also influence the statistical power [28]. Nevertheless, the observed trends, together with effect size estimates, suggest that exercise intensity (i.e., above the GET) may influence irisin regulation. Higher-intensity exercise is known to induce greater metabolic stress, increased muscle fiber recruitment, and enhanced activation of signaling pathways involved in mitochondrial biogenesis, including PGC-1α, a key regulator of FNDC5 expression and irisin release [29]. Although the POL training model includes a proportion of high-intensity exercise performed near V’O_2_max, the THR TID is characterized by a greater amount of time spent at intensities between the two ventilatory thresholds and V’O_2_max. This may result in a more sustained metabolic stimulus, potentially leading to a greater cumulative activation of pathways involved in mitochondrial biogenesis. Therefore, it is possible that the distribution and continuity of the exercise stimulus, rather than peak intensity alone, could play an important role in modulating irisin response. These mechanisms may explain the intensity-dependent modulation of irisin observed in the present study, although further research with larger sample sizes is required to confirm this hypothesis

The third important result was that, at the end of the training program, serum irisin levels were significantly and positively associated with V’O_2_RCP and V’O_2_max. These associations may support the hypothesis that irisin may reflect exercise-induced improvements in aerobic capacity [31,32]. The positive correlations between irisin and VO_2_RCP and VO_2_max suggest that individuals with greater aerobic adaptations also exhibit higher circulating irisin levels, supporting the concept that irisin may be involved in physiological adaptations to endurance training.

Indeed, several studies report that irisin promotes mitochondrial function, enhances oxidative metabolism, and facilitates energy expenditure, suggesting that higher irisin levels may be linked to improved metabolic efficiency and muscle function [33]. While the present study does not establish a causal relationship, the observed correlations indicate that irisin may represent a useful biomarker of physiological adaptations to exercise training in individuals with obesity, supported by the correlation at the end of the 24-week training. However, the cross-sectional nature of these analyses precludes the inference of the direction of causality. Thus, although irisin levels is associated with aerobic capacity, it is still unclear whether irisin contributes to these adaptations or rather reflects a baseline physiological profile; it is plausible that individuals with higher initial physical capacity respond more effectively to training.

The evaluation of irisin in saliva, alongside serum, represents a potential advantage, as saliva is a non-invasive biological fluid that allows easier, stress-free, and repeated sampling compared to blood collection. Interestingly, the group × time interaction for salivary irisin was closer to statistical significance compared to serum measurements. This observation may suggest that salivary irisin could be sensitive to training-induced changes. However, this interpretation should be approached with caution. The reduced and variable sample size for salivary analyses, together with the known variability of salivary biomarkers, limits the strength of this observation. Moreover, the lack of statistically significant changes prevents any definitive conclusion regarding the relative sensitivity of salivary versus serum irisin.

This study has several limitations that should be acknowledged. The relatively small sample size limits statistical power and may have prevented detection of significant changes in irisin concentrations. Additionally, the study included only adult males with obesity, limiting the generalizability of the findings to other populations. Additional limitations include the lack of a non-exercise control group, which limits the ability to establish causal relationships between exercise training and observed outcomes, as well as the assessment of dietary habits, which were collected on two weekdays and two holidays. Future studies with larger cohorts, inclusion of female participants, and a monitored dietary protocol are needed.

Despite these limitations, this study provides novel insights into the relationship between long-term aerobic training, exercise intensity distribution, and irisin response in individuals with obesity.

## 5. Conclusions

In conclusion, 24 weeks of POL and THR training improved body composition and cardiorespiratory fitness in male adults with obesity. Although no significant changes in irisin levels were observed at the group level, a trend toward increased irisin in the THR group and its association with aerobic fitness parameters suggest a potential link between irisin and exercise-induced adaptations. These preliminary findings support the hypothesis that irisin may reflect physiological adaptations to training, although further studies with larger cohorts are needed.

## Figures and Tables

**Table 1:** Anthropometric characteristics and irisin values before (T0), and after 24 weeks (T1) of weight management program in the pooled samples.

	All Subjects (n: 13)						*p* Value
	T0			T1			
Body mass (kg)	99.0	±	10.8	94.6	±	11.5	0.001
BMI (kg/m^−2^)	32.0	±	3.0	30.6	±	3.0	0.001
Waist (cm)	103.0	±	6.7	102.0	±	7.8	0.301
Hip (cm)	109.0	±	4.7	108.2	±	5.7	0.480
Waist-to-hip ratio	0.95	±	0.05	0.94	±	0.04	0.781
Fat-free mass (kg)	64.1	±	4.8	64.5	±	4.8	0.857
Fat Mass (kg)	34.6	±	7.2	30.1	±	7.6	0.001
Fat-free mass (%)	65.2	±	3.9	68.6	±	4.2	0.001
Fat Mass (%)	35.0	±	3.9	31.5	±	4.2	0.001
Adiponectin (mg/mL)	22.3	±	4.5	27.0	±	2.6	0.001
Salivary Adiponectin (ng/mL)	17.6	±	7.5	22.8	±	10.0	0.005
Serum Irisin (ng/mL)	10.2	±	8.0	11.0	±	7.9	0.737
Salivary Irisin (ng/mL)	0.69		(6.71)	0.86		(6.23)	0.850

All values are presented as mean ± standard deviation or median and interquartile range. BMI: body mass index.

**Table 2:** Physiological Parameters before (T0) and after 24 weeks (T1) of weight management program in the pooled samples.

	All Subjects (n: 13)						*p* Value
	T0			T1			
**Maximal oxygen uptake**							
V’O_2_ (L min^−1^)	3.89	±	0.39	4.07	±	0.44	0.025
V’O_2_ (mL/kg/min)	39.6	±	4.4	43.3	±	6.4	0.015
HRmax (bpm)	180.2	±	7.7	175.4	±	9.6	0.040
RER max	1.10	±	0.06	1.09	±	0.04	0.567
**Respiratory compensation point**							
V’O_2_ (L min^−1^)	3.50	±	0.35	3.62	±	0.44	0.186
V’O_2_ (mL/kg/min)	35.6	±	4.3	38.7	±	6.0	0.008
V’O_2_, (%max)	89.9	±	3.3	89.3	±	5.4	0.743
HR (bpm)	167.2	±	8.6	165.0	±	10.2	0.287
HR, %max	93.0	±	3.6	93.8	±	2.6	0.405
RER	1.00	±	0.04	0.99	±	0.04	0.820
**Gas exchange threshold**							
V’O_2_ (L min^−1^)	2.74	±	0.46	3.02	±	0.42	0.008
V’O_2_ (mL/kg/min)	28.2	±	6.4	32.4	±	6.0	0.001
V’O_2_, %max	70.5	±	10.4	75.0	±	8.6	0.107
HR (bpm)	146.5	±	14.0	147.4	±	11.6	0.822
HR (%max)	81.2	±	6.1	84.1	±	6.4	0.201
RER	0.91	±	0.06	0.91	±	0.04	0.175

All values are presented as mean ± standard deviation. V’O_2_: oxygen consumption, HR: heart rate, RER: respiratory exchange ratio, V’O_2_ %max: percentage of maxima oxygen uptake, HR %max: percentage of heart rate max.

**Table 3:** Daily energy and macronutrient intake before (T0), and after 24 weeks (T1) of weight management program in the pooled samples.

	All Subjects (n: 13)						*p* Value
	T0			T1			
Total EE (kcal/day^−1^)	1662.0	±	329.4	1722.2	±	376.2	0.242
EE from CHO (kcal/day^−1^)	721.2	±	282.9	757.1	±	233.7	0.212
EE from Fat (kcal/day^−1^)	579.8	±	111.6	580.9	±	150.9	0.763
EE from Protein (kcal/day^−1^)	292.5	±	63.6	108.2	±	57.8	0.879

All values are presented as mean ± standard deviation. EE: energy expenditure, CHO: carbohydrate.

## Data Availability

The original contributions presented in this study are included in the article/Supplementary Materials. Further inquiries can be directed to the corresponding author.

## Supplementary Materials

The following supporting information can be downloaded at: https://www.mdpi.com/article/10.3390/life16040657/s1; Tables S1 and S2. Supplementary Table S1. Correlations between Serum Irisin and Salivary Irisin with the anthropometric parameters and adiponectin values at the end of the 24 weeks of training (T1). Supplementary Table S2. Correlations between Serum Irisin and Salivary Irisin with the physical capacities at the end of the 24 weeks of training (T1).

## References

[1] Koliaki C., Dalamaga M., Liatis S. (2023). Update on the Obesity Epidemic: After the Sudden Rise, Is the Upward Trajectory Beginning to Flatten?. Curr. Obes. Rep..

[2] World Obesity Federation Italy—Country Profile. https://data.worldobesity.org/country/italy-102/.

[3] Solmi M., Basadonne I., Bodini L., Rosenbaum S., Schuch F.B., Smith L., Stubbs B., Firth J., Vancampfort D., Ashdown-Franks G. (2025). Exercise as a transdiagnostic intervention for improving mental health: An umbrella review. J. Psychiatr. Res..

[4] WHO (2021). Physical Activity Fact Sheet—Italy 2021.

[5] Higuera-Hernández M.F., Reyes-Cuapio E., Gutiérrez-Mendoza M., Rocha N.B., Veras A.B., Budde H., Jesse J., Zaldívar-Rae J., Blanco-Centurión C., Machado S. (2018). Fighting obesity: Non-pharmacological interventions. Clin. Nutr. ESPEN.

[6] Jakicic J.M., Apovian C.M., Barr-Anderson D.J., Courcoulas A.P., Donnelly J.E., Ekkekakis P., Hopkins M., Lambert E.V., Napolitano M.A., Volpe S.L. (2024). Physical activity and excess body weight and adiposity for adults. Med. Sci. Sports Exerc..

[7] MacInnis M.J., Gibala M.J. (2017). Physiological adaptations to interval training and the role of exercise intensity. J. Physiol..

[8] Vaccari F., Passaro A., D’Amuri A., Sanz J.M., Di Vece F., Capatti E., Magnesa B., Comelli M., Mavelli I., Grassi B. (2020). Effects of 3-month high-intensity interval training vs. moderate endurance training and 4-month follow-up on fat metabolism, cardiorespiratory function and mitochondrial respiration in obese adults. Eur. J. Appl. Physiol..

[9] D’Alleva M., Vaccari F., Graniero F., Giovanelli N., Floreani M., Fiori F., Marinoni M., Parpinel M., Lazzer S. (2023). Effects of 12-week combined training versus high intensity interval training on cardiorespiratory fitness, body composition and fat metabolism in obese male adults. J. Exerc. Sci. Fit..

[10] D’Alleva M., Giovanelli N., Graniero F., Billat V.L., Fiori F., Marinoni M., Parpinel M., Lazzer S. (2024). Effects of 24-week Polarized Training vs. Threshold Training in Obese Male Adults. Int. J. Sports Med..

[11] Bao J.F., She Q.Y., Hu P.P., Jia N., Li A. (2022). Irisin, a fascinating field in our times. Trends Endocrinol. Metab..

[12] Mahgoub M.O., D’Souza C., Al Darmaki R.S.M.H., Baniyas M.M.Y.H., Adeghate E. (2018). An update on the role of irisin in the regulation of endocrine and metabolic functions. Peptides.

[13] Kim S.H., Kim S.E., Kim S., Ahn M.B., Cho W.K., Cho K.S., Jung M.H. (2024). The association of serum irisin with anthropometric, metabolic, and bone parameters in obese children and adolescents. Front. Endocrinol..

[14] Jia J., Yu F., Wei W.P., Yang P., Zhang R., Sheng Y., Shi Y.-Q. (2019). Relationship between circulating irisin levels and overweight/obesity: A meta-analysis. World J. Clin. Cases.

[15] Hejazi J., Ghobadian B., Ghasemi N., Sadeh H., Abedimanesh N., Rahimlou M. (2025). Relationship of serum irisin levels, physical activity, and metabolic syndrome biomarkers in obese individuals with low-calorie intake and non-obese individuals with high-calorie intake. J. Health Popul. Nutr..

[16] Kim H.J., So B., Choi M., Kang D., Song W. (2015). Resistance exercise training increases the expression of irisin concomitant with improvement of muscle function in aging mice and humans. Exp. Gerontol..

[17] D’Amuri A., Raparelli V., Sanz J.M., Capatti E., Di Vece F., Vaccari F., Lazzer S., Zuliani G., Dalla Nora E., Neri L.M. (2022). Biological Response of Irisin Induced by Different Types of Exercise in Obese Subjects: A Non-Inferiority Controlled Randomized Study. Biology.

[18] Riahy S. (2024). The effects of 12 weeks of high-intensity interval training and moderate-intensity continuous training on FGF21, irisin, and myostatin in men with type 2 diabetes mellitus. Growth Factors.

[19] Seiler S. (2010). What is best practice for training intensity and duration distribution in endurance athletes?. Int. J. Sports Physiol. Perform..

[20] Campos Y., Casado A., Vieira J.G., Guimarães M., Sant’Ana L., Leitao L., da Silva S.F., de Azevedo P.H.S.M., Vianna J., Domínguez R. (2022). Training-intensity distribution on middle- and long-distance runners: A systematic review. Int. J. Sports Med..

[21] Mallardo M., D’Alleva M., Lazzer S., Giovanelli N., Graniero F., Billat V., Fiori F., Marinoni M., Parpinel M., Daniele A. (2023). Improvement of adiponectin in relation to physical performance and body composition in young obese males subjected to twenty-four weeks of training programs. Heliyon.

[22] Kagawa M., Byrne N.M., Hills A.P. (2008). Comparison of body fat estimation using waist:height ratio using different ‘waist’ measurements in Australian adults. Br. J. Nutr..

[23] Gray D.S., Bray G.A., Gemayel N., Kaplan K. (1989). Effect of obesity on bioelectrical impedance. Am. J. Clin. Nutr..

[24] Missaglia S., Tommasini E., Vago P., Pecci C., Galvani C., Silvestrini A., Mordente A., Tavian D. (2023). Salivary and serum irisin in healthy adults before and after exercise. Eur. J. Transl. Myol..

[25] Hecksteden A., Wegmann M., Steffen A., Kraushaar J., Morsch A., Ruppenthal S., Kaestner L., Meyer T. (2013). Irisin and exercise training in humans—Results from a randomized controlled training trial. BMC Med..

[26] Cohen J. (1988). Statistical Power Analysis for the Behavioral Sciences.

[27] Srivastava S., Tamrakar S., Nallathambi N., Vrindavanam S.A., Prasad R., Kothari R. (2024). Assessment of Maximal Oxygen Uptake (VO2 Max) in Athletes and Nonathletes Assessed in Sports Physiology Laboratory. Cureus.

[28] Zeyu W., Preobrazenski N., Renwick J.R.M., Khansari A., LeBouedec M.A., Nuttall J.M.G., Mudwi A., Ross B., Simpson-Stairs N., Beaupre L.P.R. (2026). Aerobic Exercise Training and VO_2max_: A Scoping Review of Study Populations and Protocols. J. Funct. Morphol. Kinesiol..

[29] Newman J.E., King I., Flemming N., Broadhouse K.M., Buhmann R., Rose G.L., Jenkins D.G., Askew C.D., Schaumberg M.A. (2025). The acute response of irisin to resistance and endurance exercise at both lower and higher intensities in healthy older adults. Exp. Gerontol..

[30] Curovic I. (2025). The role of resistance exercise-induced local metabolic stress in mediating systemic health and functional adaptations: Could condensed training volume unlock greater benefits beyond time efficiency?. Front. Physiol..

[31] Almeida González D., Rodríguez-Pérez M.D.C., Fuentes Ferrer M., Cuevas Fernández F.J., Marcelino Rodríguez I., Cabrera de León A. (2023). Irisin, in women and men: Blood pressure, heart rate, obesity and insulin resistance. Front. Endocrinol..

[32] Boström P., Wu J., Jedrychowski M.P., Korde A., Ye L., Lo J.C., Rasbach K.A., Boström E.A., Choi J.H., Long J.Z. (2012). A PGC1-α-dependent myokine that drives brown-fat-like development of white fat and thermogenesis. Nature.

[33] Colpitts B.H., Rioux B.V., Eadie A.L., Brunt K.R., Sénéchal M. (2022). Irisin response to acute moderate intensity exercise and high intensity interval training in youth of different obesity statuses: A randomized crossover trial. Physiol. Rep..

