# Supplementary material for: Effects of Two Different Training Programs on Cardiometabolic Health, Body Composition and Irisin in Middle Age Obese Males: A Pilot Study
Source: Life (Basel). 2026 Apr 13;16(4):657. doi: 10.3390/life16040657 (PMC13117881; doi:10.3390/life16040657)
Supplement: Supplementary file 1 [file life-16-00657-s001.zip › life-4212935-supplementary.pdf]

Supplementary Table S1. Correlations between irisin and salivary irisin with the anthropometric parameters and adiponectin values at baseline (T0).

	BM (kg)	BMI (kg/m^{-2})	FFM (kg)	FM (kg)	FFM (%)	FM (%)	Waist (cm)	Hip (cm)	Waist-to-hip ratio	Adiponectin ($\mu g/ml^{-1}$)	Salivary adiponectin (ng/ml^{-1})
Irisin (ng/ml^{-1})	r = 0.292<br>P = 0.357	r = 0.092<br>P = 0.776	r = 0.024<br>P = 0.941	r = 0.404<br>P = 0.193	r = -0.434<br>P = 0.157	r = 0.436<br>P = 0.157	r = 0.346<br>P = 0.271	**r = 0.576**<br>**P = 0.048**	r = 0.040<br>P = 0.908	r = -0.100<br>P = 0.758	r = -0.265<br>P = 0.406
Salivary Irisin (ng/ml^{-1})	R = -0.098<br>P = 0.792	R = -0.210<br>P = 0.554	R = -0.183<br>P = 0.610	R = -0.280<br>P = 0.431	R = 0.232<br>P = 0.513	R = -0.232<br>P = 0.513	R = -0.079<br>P = 0.830	R = 0.154<br>P = 0.672	R = -0.083<br>P = 0.820	R = 0.219<br>P = 0.542	R = -0.457<br>P = 0.184

BM: body mass, BMI: body mass index, FFM: fat free mass, FM: fat mass.
Bold text indicates a statistically significant correlation
r value obtained with Pearson's correlation coefficients.
R value obtained with Spearman's correlation coefficients (non-normally distributed data).
Bold text indicates a statistically significant correlation.

Supplementary Table S2. Correlations between irisin and salivary irisin with the physical capacities at baseline (T0).

	V'O_2 GET (L/min^{-1})	V'O_2 RCP (L/min^{-1})	V'O_2max (L/min^{-1})	V'O_2 GET ($ml/kg^{-1}/min^{-1}$)	V'O_2 RCP ($ml/kg^{-1}/min^{-1}$)	V'O_2max ($ml7kg^{-1}/min^{-1}$)	V_{GET} (km/h^{-1})	V_{RCP} (km/h^{-1})	V_{MAX} (km/h^{-1})	V'O_2GET (%max)	V'O_2RCP (%max)
Irisin ($ng\ ml^{-1}$)	r = -0.188<br>P = 0.559	r = 0.090<br>P = 0.783	r = 0.277<br>P = 0.383	r = -0.225<br>P = 0.483	r = -0.159<br>P = 0.622	r = -0.022<br>P = 0.945	r = -0.246<br>P = 0.441	r = -0.205<br>P = 0.522	r = -0.250<br>P = 0.433	r = -0.346<br>P = 0.271	r = -0.483<br>P = 0.112
Salivary Irisin (ng/ml^{-1})	R = 0.054<br>P = 0.884	R = -0.219<br>P = 0.542	R = -0.170<br>P = 0.542	R = -0.030<br>P = 0.938	R = 0.103<br>P = 0.772	R = 0.195<br>P = 0.589	R = 0.248<br>P = 0.486	R = 0.071<br>P = 0.846	R = 0.078<br>P = 0.831	R = 0.048<br>P = 0.899	R = -0.204<br>P = 0.566

V'O_2: oxygen consumption, RCP: respiratory compensation point, GET: gas exchange threshold, V: velocity
Bold text indicates a statistically significant correlation
r value obtained with Pearson's correlation coefficients.
R value obtained with Spearman's correlation coefficients (non-normally distributed data).
Bold text indicates a statistically significant correlation.
